# Heart Failure Associated with Giant Uterine Leiomyoma: A Case Report

**DOI:** 10.3390/medicina60111892

**Published:** 2024-11-18

**Authors:** Hai-Ning Hsu, Fang-Chin Hsu, Yuan Hung, Po-Chao Hsu, Kuo-Min Su

**Affiliations:** 1 Department of Obstetrics and Gynecology, Tri-Service General Hospital, National Defense Medical Center, No. 325, Sec. 2, Chenggong Rd., Neihu District, Taipei City 11490, Taiwan; 2 Department of Surgery, Tri-Service General Hospital, National Defense Medical Center, No. 325, Sec. 2, Chenggong Rd., Neihu District, Taipei City 11490, Taiwan; 3 Division of Cardiology, Department of Internal Medicine, Taipei Medical University Hospital, No. 250, Wuxing St., Xinyi District, Taipei City 11042, Taiwan; 4 Division of Cardiology, Department of Internal Medicine, Tri-Service General Hospital, National Defense Medical Center, No. 325, Sec. 2, Chenggong Rd., Neihu District, Taipei City 11490, Taiwan

**Keywords:** fibroid, myoma, angiogenesis, estrogen

## Abstract

Heart failure impairs the heart’s pumping ability and triggers catecholamine production as an adaptive mechanism. Uterine leiomyomas are common tumors of the female reproductive tract. Their growth is promoted by dysregulated angiogenesis and gonadal steroid hormones. Although uterine leiomyomas share risk factors with most cardiovascular diseases, their relationship with heart failure has not been well described. Herein, we present the case of a 45-year-old woman with heart failure who visited the emergency department, where we incidentally discovered a giant uterine leiomyoma. The patient was admitted with progressive dyspnea and abdominal distension. Echocardiography revealed an enlarged right ventricle and a decreased systolic function. Computed tomography revealed cardiomegaly with bilateral pleural effusions and a tumor measuring 18.0 × 12.0 cm in the abdominal cavity with massive ascites. A diagnosis of heart failure in conjunction with a uterine leiomyoma was established, which prompted the prescription and adjustment of heart failure medications according to the patient’s clinical presentation. Three weeks later, given the persistent symptoms of bilateral lower extremities pitting edema and abdominal distension, a total hysterectomy was performed. Postoperatively, echocardiography revealed marked improvement in her heart failure. The patient was discharged in a stable clinical and hemodynamic conditions, and reported good physical condition at the 4-month follow-up. Growth factors and the compression effect of uterine leiomyomas may predispose patients to heart failure and exacerbate its deterioration. Although reports of fibroid-related heart failure are rare, uterine leiomyomas should be considered a potential cause of refractory heart failure. Nevertheless, a direct association requires a longer follow-up period.

## 1. Introduction

Heart failure is the progressive deterioration in the heart’s ability to pump blood, accompanied by a series of cellular, structural, and neurohumoral changes following cardiac abnormalities [1]. Key contributors to this process include the activation of the sympathoadrenergic and renin–angiotensin–aldosterone systems [1]. Patients with heart failure may present with a variety of signs, such as elevated jugular venous pressure and peripheral edema, along with symptoms like dyspnea and ankle swelling [2]. Cardiovascular diseases share several risk factors with uterine leiomyomas (fibroids or myomas) [3,4]. Fibroids are the most common benign neoplasms of the female reproductive system, primarily affecting women of childbearing age [5]. However, only one in five patients with this condition present with irregular bleeding, anemia, and pelvic pain [6]. Fibroid growth depends on vascular support and gonadal steroid hormones [6,7], and may induce a compression effect on nearby structures. These factors may contribute to worsening heart failure. Notably, while cases of heart failure due to benign metastasizing leiomyoma (BML) have been described [8,9,10], reports of fibroid-related heart failure are rare. Therefore, this case report aims to investigate the potential link between uterine leiomyomas and heart failure, particularly in cases where cardiac causes are absent.

In this case report, we describe the case of a patient with heart failure. Upon examination, we incidentally discovered a giant fibroid. Apparent improvements in atrial and ventricular size and systolic function after total hysterectomy were observed in the patient.

## 2. Case Presentation

**Figure 1:**
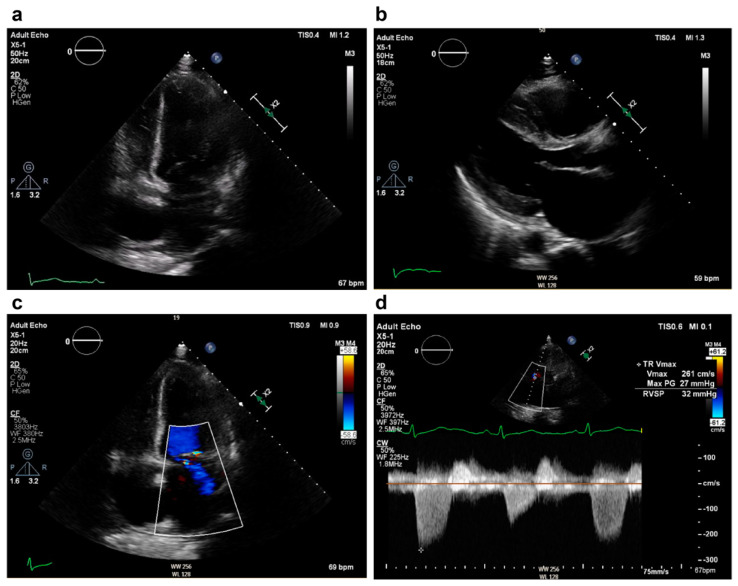
Preoperative echocardiographic findings. (**a**) Apical four-chamber view in end-diastolic state showing generalized hypokinesia of the left ventricle with a left ventricular ejection fraction of 20%. (**b**) Parasternal long axis view showing left atrial dilation. (**c**) Apical four-chamber view showing moderate mitral regurgitation. (**d**) Severe tricuspid regurgitation with elevated pulmonary artery systolic pressure of 32 mmHg.

**Figure 2:**
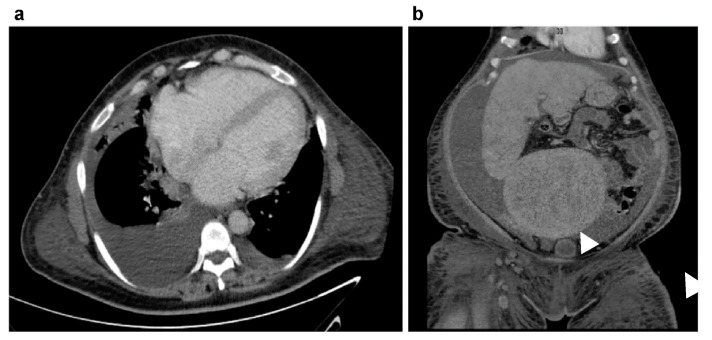
Computed tomography (CT) scan. (**a**) Chest CT reveals bilateral pleural effusions. (**b**) Abdominal and pelvic CT demonstrate a uterine tumor (arrowhead) measuring 18.0 × 12.0 cm.

**Figure 3:**
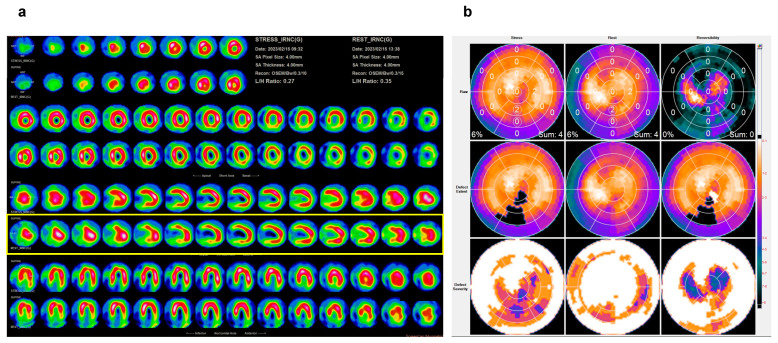
Thalium-201 myocardial perfusion scans. (**a**) The scan revealed delayed-onset moderate hypoperfusion over the apical and anterior walls of the left ventricle (yellow frame). (**b**) The 17 segment model revealed mild-to-moderate myocardial ischemia injury involving the right coronary and left anterior descending arteries.

A nulligravid 45-year-old woman visited the emergency department because of abdominal bloating, which had been occurring for several months, and exacerbated dyspnea, which started 2 weeks before her visit to our hospital. She had experienced amenorrhea for the past 4 months, but this had not been investigated. The patient denied any history of systemic disease. Physical examination revealed a body mass index of 39.2 kg/m^2^, blood pressure of 159/98 mmHg, heart rate of 116 beats/min, lower extremity pitting edema, and diffuse abdominal tenderness. Laboratory results included hemoglobin at 9.0 g/dL, pro-B-type natriuretic peptide (pro-BNP) at 5616.0 pg/mL, creatine kinase at 386 U/L, troponin I at 54 pg/mL, aspartate transaminase at 85 U/L, and alanine transaminase at 112 U/L. Electrocardiographic findings were normal, except for sinus tachycardia. Transthoracic echocardiography revealed moderate mitral and severe tricuspid regurgitation. The right ventricle (RV) was enlarged. The left ventricle (LV) dimensions were normal, with generalized hypokinesia, and depressed systolic function (Figure 1). Computed tomography revealed cardiomegaly with massive bilateral pleural effusions and a giant soft-tissue mass measuring 18.0 × 12.0 cm located in the abdominal cavity with massive ascites (Figure 2). BML was ruled out. The patient reported daily use of valsartan (160 mg), spironolactone (25 mg), and bisoprolol (1.25 mg). An intravenous transfusion of furosemide (20 mg) once a day was administered on the 10th day of admission because of persistent bilateral lower extremity edema and dyspnea. A thallium-201 (Tl-201) myocardial perfusion scan revealed delayed-onset moderate hypoperfusion over the apical and anterior walls of the LV and mild-to-moderate myocardial ischemic injury involving the right coronary and left anterior descending arteries (Figure 3).

**Figure 4:**
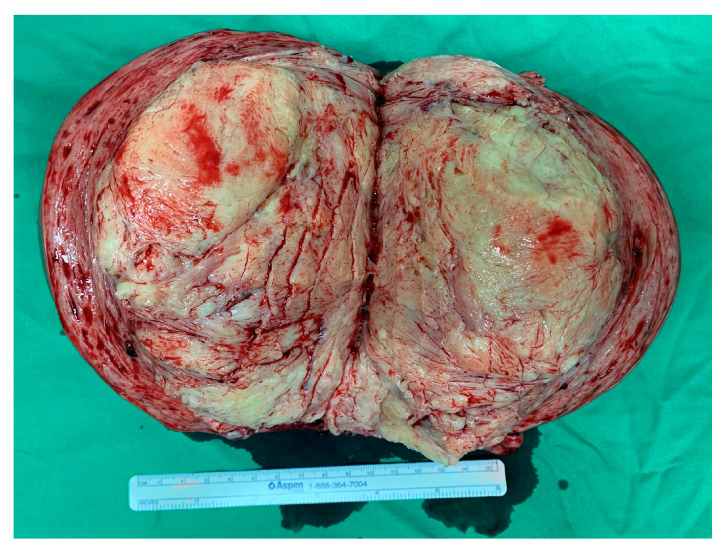
Photograph of excised mass. An 18.0 × 12.0 cm myoma of the uterus was excised during a total hysterectomy. The length of the pictured ruler is 15 cm.

**Figure 5:**
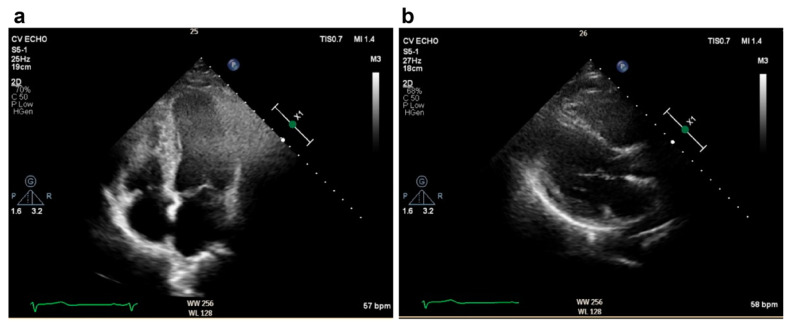
Postoperative echocardiographic findings. (**a**) Apical four-chamber view in an end-diastolic state showing a normal-sized left ventricle with a left ventricular ejection fraction of 68%. (**b**) Parasternal long axis view showing normal RV and LV size.

For a differential diagnosis of occlusive coronary artery disease, the patient underwent coronary angiography, which revealed normal coronary arteries. Myocarditis and cardiomyopathy were not significantly associated with serum examination, electrocardiography, or echocardiography findings. Aggravating factors of heart failure, such as sepsis, fluid overload, arrhythmia, and hypertension, were investigated and treated, respectively. Specifically, the patient’s vital signs, liver function, and renal function were closely monitored. Bacteria cultures of blood, urine, ascites fluid, and pleural fluid all yielded negative findings. Repeated EKG conducted during hospitalization revealed no findings of arrhythmia. Fluid overload and hypertension were controlled with medications. Cardiac ascites was diagnosed by peritoneal fluid analysis, showing a serum ascites albumin gradient (1.3 g/dL) and ascitic protein (2.8 g/dL), along with elevated pro-BNP levels. Further evaluation with an echocardiogram and echocardiography confirmed the diagnosis. Three weeks later, the patient’s dyspnea was alleviated but bilateral lower extremity pitting edema and abdominal distension persisted; therefore, a total hysterectomy was performed (Figure 4). Histopathological examination revealed cellular uterine leiomyoma. One month later, there was significant resolution of the pitting edema, abdominal tenderness, pleural effusion, and ascites. Additionally, pro-BNP levels declined from 5616 pg/mL to 377 pg/mL. Postoperative echocardiography revealed satisfactory mitral and tricuspid valve function, normal RV and LV size, and an improved LV systolic function, with an ejection fraction at 68% (Figure 5). The patient was discharged from the hospital in stable clinical and hemodynamic conditions. Her medications were reviewed, and she was prescribed bisoprolol (1.25 mg), dapagliflozin (10 mg), furosemide (40 mg), spironolactone (25 mg), and sacubitril/valsartan (50 mg) BID. Furosemide and spironolactone were discontinued 2 months after hospital discharge. Four months after hospital discharge, the dose of bisoprolol was tapered to half, and the patient reported having a good physical condition and did not have any complications.

## 3. Discussion

Fibroids develop in size with dysregulated angiogenesis [6]. Tumors secrete angiogenic factors to increase vascular density in the surrounding normal myometrium, followed by the vascularized capsule surrounding the avascular core [6]. This, in turn, may induce abnormal uterine bleeding and a tendency to bleed during myomectomy, which was also observed in our case [6,11,12].

Gonadal steroid hormones, predominantly estrogen, contribute to both the development of fibroids and resistance to heart failure [13,14]. Estrogen induces the proliferation of uterine tissue and fibroids by increasing the expression of multiple angiogenic factors in myometrial and leiomyoma tissues by binding to the estrogen receptor (ER)α, whereas ERβ dominates the cardiovascular system to stimulate antioxidative and anti-inflammatory activities [14,15]. When ERβ is co-expressed with ERα, it demonstrates an opposing reaction to ERα [16]. Protection against heart failure may decrease owing to higher levels of tissue estrogen and estrogen receptors in fibroid cells compared to normal myometrium [14,15,17]. Both irregular menstrual cycles and menopause are associated with significant changes in hormonal patterns, which cause increased risk of heart failure [18,19]. In patients with heart failure with reduced ejection fraction, elevated estradiol and dehydroepiandrosterone levels, along with a lower total testosterone/estradiol ratio, have been associated with a reduced risk of heart failure [20]. In our case, the patient experienced menstrual irregularities for 4 months, suggesting the onset of perimenopause, which is known to be a period of accelerated risk of cardiovascular disease [18,19]. However, a limitation of this case is the lack of investigation into the patient’s sex hormone levels.

The compression caused by fibroids induces acute complications, including thromboembolism, acute urinary retention, renal failure, mesenteric vein thrombosis, and intestinal gangrene [21]. Resuscitation in the left decubitus position or emergency laparotomy is considered for relief [22]. However, decreased preload due to compression of the inferior vena cava may cause heart damage and, in the long run, induce heart failure. Cardiac arrest, although rare, has been reported as a fatal complication of extremely large fibroids [22].

In addition, a higher demand for blood supply by fibroids leads to myocardial ischemia and anemia, which cause an initial decline in the pumping capacity of the heart. In response, compensatory mechanisms, including catecholamines, are activated to increase cardiac contractility and myocardial oxygen demand in the long term, which may induce life-threatening arrhythmias and activate the signaling pathways of hypertrophy and cell death [1]. Thus, heart failure is aggravated in this vicious cycle.

In our case, the rapid preoperative increase in LV ejection fraction during the stress test conducted 1 week prior to surgery was attributed to increased muscle sympathetic nerve activity in heart failure with reduced ejection fraction, as well as the effects of medication. However, prolonged neurohumoral activation caused anemia and myocardial ischemia in our patient, as revealed by a Tl-201 myocardial perfusion scan.

BMLs with uncommon metastasis sites in the heart are known to potentially induce heart disease, including heart failure [8,9,10]; however, fibroid-related heart failure is rarely reported.

While this report offers valuable insights into the association between uterine leiomyomas and heart failure, some limitations should be acknowledged. This is a retrospective case report involving a single patient, and further studies with larger sample size and longer follow-up are needed. Nonetheless, fibroids should be considered as a differential diagnosis in female patients without a history of fibroids, especially whose refractory heart failure does not have an evident cardiac cause.

## 4. Conclusions

This report suggests that increased demand for blood supply, the dominant effect of ERα compared to that of ERβ, and compression complications of fibroids may theoretically contribute to the diagnosis of heart failure. Furthermore, long-term catecholamine activation can aggravate heart failure. Fibroids should be considered as a potential cause of refractory heart failure, warranting a multidisciplinary approach for investigation and management. However, a direct association between fibroids and heart failure has not been confirmed, necessitating large-scale cohort studies with longer follow-up periods for a more comprehensive evaluation.

## Data Availability

Data sharing is not applicable as no new data were created or analyzed in this study.
